# Efficacy of melatonin in decreasing the incidence of *delirium* in critically ill adults: a randomized controlled trial

**DOI:** 10.62675/2965-2774.20240144-en

**Published:** 2024-04-09

**Authors:** Anjishnujit Bandyopadhyay, Lakshmi Narayana Yaddanapudi, Vikas Saini, Neeru Sahni, Sandeep Grover, Sunaakshi Puri, Vighnesh Ashok

**Affiliations:** 1 All India Institute of Medical Sciences Jai Prakash Narayan Apex Trauma Center Pain Medicine and Critical Care New Delhi India Department of Anaesthesiology, Pain Medicine and Critical Care, Jai Prakash Narayan Apex Trauma Center, All India Institute of Medical Sciences - New Delhi, India.; 2 Nehru Hospital Institute of Medical Education and Research Department of Anaesthesia and Intensive Care, Level 4 Chandigarh India Department of Anaesthesia and Intensive Care, Level 4, Nehru Hospital, Postgraduate Institute of Medical Education and Research, Sector 12 - Chandigarh, India.; 3 Nehru Hospital Institute of Medical Education and Research Department of Psychiatry Chandigarh India Department of Psychiatry, Nehru Hospital, Postgraduate Institute of Medical Education and Research, Sector 12 - Chandigarh, India.

**Keywords:** Circadian rhythm, Critically illness, Delirium, Melatonin, Sleepwake disorders, Intensive care units

## Abstract

**Objective:**

To determine whether enteral melatonin decreases the incidence of *delirium* in critically ill adults.

**Methods:**

In this randomized controlled trial, adults were admitted to the intensive care unit and received either usual standard care alone (Control Group) or in combination with 3mg of enteral melatonin once a day at 9 PM (Melatonin Group). Concealment of allocation was done by serially numbered opaque sealed envelopes. The intensivist assessing *delirium* and the investigator performing the data analysis were blinded to the group allocation. The primary outcome was the incidence of *delirium* within 24 hours of the intensive care unit stay. The secondary outcomes were the incidence of *delirium* on Days 3 and 7, intensive care unit mortality, length of intensive care unit stay, duration of mechanical ventilation and Glasgow outcome score (at discharge).

**Results:**

We included 108 patients in the final analysis, with 54 patients in each group. At 24 hours of intensive care unit stay, there was no difference in the incidence of *delirium* between Melatonin and Control Groups (29.6 *versus* 46.2%; RR = 0.6; 95%CI 0.38 - 1.05; p = 0.11). No secondary outcome showed a statistically significant difference.

**Conclusion:**

Enteral melatonin 3mg is not more effective at decreasing the incidence of *delirium* than standard care is in critically ill adults.

## INTRODUCTION

*Delirium* is an acute confusional state characterized by fluctuating disturbances in cognition and attention. It is known to be associated with the dysregulation of dopaminergic, cholinergic, gamma amino butyric acidergic (GABAergic), serotonergic and catecholinergic neurotransmission.^(1)^ The prevalence of *delirium* ranges between 11 and 83% in patients admitted to the intensive care unit (ICU).^(2,3)^ *Delirium* increases the risk of mortality, length of ICU stay, and hospital costs and hence warrants as much attention as other organ failures in the ICU.^(4)^

The etiology of *delirium* is multifactorial, and in an ICU setting, disturbance of the circadian rhythm and sleep cycle can be major contributing factors. Sleep deprivation in the ICU occurs due to environmental disturbances, loss of diurnal routine, critical illness itself or treatment being administered to the patients.^(5)^

The rationale for the use of melatonin for decreasing sleep disturbances and potentially *delirium* in the ICU is derived from the finding of low plasma melatonin concentrations and abnormal patterns of secretion of melatonin in critically ill patients.^(6)^ However, a definite prophylactic/therapeutic effect of melatonin has not yet been established, as is evident by the contrary findings reported in systematic reviews and meta-analyses (SRMAs) published by Yan et al. and Aiello et al.^(7,8)^

In the present study, we evaluated whether prophylactic/therapeutic melatonin decreases the incidence of *delirium* compared with standard care in critically ill adults admitted to the ICU. The duration of mechanical ventilation (MV), length of ICU stay, mortality and Glasgow outcome scale score at discharge were studied as secondary outcomes. Therefore, our objective was to determine whether enteral melatonin decreases the incidence of delirium in critically ill adults.

## METHODS

This was an open-label parallel group randomized controlled trial conducted in a mixed medical-surgical ICU of a tertiary care teaching hospital. The study was conducted between January 2020 and October 2020 after registration in the Clinical Trials Registry of India (CTRI/2020/01/023038) and approval from the institutional Ethics Committee. All patients between 18 and 65 years of age with an expected ICU stay of more than 24 hours were screened for inclusion. Written informed consent was obtained from patients/patients’ guardians before recruitment into the trial. Pregnant patients, patients who were already receiving melatonin therapy, comatose patients who could not be assessed for *delirium*, coronavirus disease 2019 (COVID-19) patients, patients who were already receiving antipsychotic medications, patients with Alzheimer's disease or Parkinson's disease and those for whom enteral drug administration was contraindicated were excluded from the study.

Patients were randomly allocated to one of the 2 groups using computer-generated permuted block randomisation. Concealment of allocation was done by serially numbered opaque sealed envelopes. Treatment was revealed only when the patient had been recruited into the trial and before the first administration of the drug. The intensivist assessing *delirium* and the investigator performing the data analysis were blinded to the group allocation.

Melatonin Group (Group M) patients received 3mg of melatonin enterally at 9 PM for 7 consecutive days in addition to standard care or until discharge from the ICU, whichever was earlier. Control Group (Group C) patients received standard care alone. The usual standard care for the prevention of *delirium* comprises the avoidance of unnecessary sedation and restraints, sedation holidays, increased family interaction, early mobility and exercise, appropriate pain management and prevention of hypoxia and dyselectrolytaemia. The use of sedo-analgesia was left to the discretion of an on-duty intensivist, but as a protocol of the ICU, the use of intravenous benzodiazepines was avoided. The commonly used sedo-analgesia drugs in our ICU are dexmedetomidine and fentanyl. The sedation infusion was targeted to a Richmond Agitation Sedation Scale (RASS) score of 0 to 1.

Immediately after admission and successful recruitment into the trial, baseline data, which included age, sex, diagnosis at the time of admission, Glasgow coma scale score, Acute Physiology and Chronic Health Evaluation (APACHE II) score and serum electrolytes (sodium, potassium, calcium and magnesium), were collected. Sequential Organ Failure Assessment (SOFA) scores, serum electrolytes and numerical rating scores (NRS) for pain were measured daily.

An assessment of *delirium* was performed via the confusion assessment method in the ICU (CAM-ICU) by an intensivist not participating in the study. The CAM-ICU assesses patients for 4 features: mental status fluctuations, inattention, altered level of consciousness and disorganized thinking.^(9)^ In the case of acute agitation, when there was a risk of extubation or weaning failure, intravenous (IV) haloperidol at a dose of 0.5mg was used every 30 minutes as a rescue agent until agitation settled or a dose of 2.5mg was reached, at which point 2mg of lorazepam was administered IV. If the patient continued to be agitated, an emergency psychiatric consultation was sought. Patients who developed *delirium* after the 7-day period were treated with 0.25mg of IV haloperidol twice a day as per the ICU protocol. Patients were followed up daily, and the following data were recorded: duration of MV, length of ICU stay, need for and dose of antipsychotics/sedatives, adverse effects, if any, due to melatonin, mortality and Glasgow outcome scale.

The primary outcome was the incidence of *delirium* (proportion of patients who were delirious at assessment) within 24 hours of the ICU stay in both groups. The secondary outcomes were the incidence of *delirium* at Days 3 and 7 of the ICU stay; incidence of *delirium* (new-onset *delirium*) at 24 hours; Day 3 and Day 7; mortality; length of ICU stay; length of MV; and Glasgow outcome scale score at discharge.

A previous study performed in our ICU showed a 68% prevalence and 60% incidence of *delirium*.^(10)^ Sample size calculation was performed on www.openepi.com. To achieve a 50% decrease in the prevalence of *delirium* in the treatment group, 49 patients were recruited in each group with a 90% power and an alpha error of 5%. We aimed to recruit a total of 126 patients in each group, accounting for 25% of the dropouts during the trial period. Categorical data such as prevalence and mortality were analyzed using the chi-square test or Fisher's exact test and are presented as risk ratios with 95% confidence intervals (95%CIs). The normality of the data was analyzed using the Shapiro–Wilk test. Data following a Gaussian distribution, such as age, are depicted as the mean with standard deviation and were analyzed with Student's t test. Non-Gaussian data such as length of ICU stay, MV, and APACHE II score are presented as medians with interquartile ranges and were analyzed using the Mann–Whitney U test. All tests were 2 tailed with a 95%CI and a level of significance of 5% (p < 0.05). Analysis was performed using Jamovi version 1.6 based on R Core version 4.0.

## RESULTS

**Figure 1:**
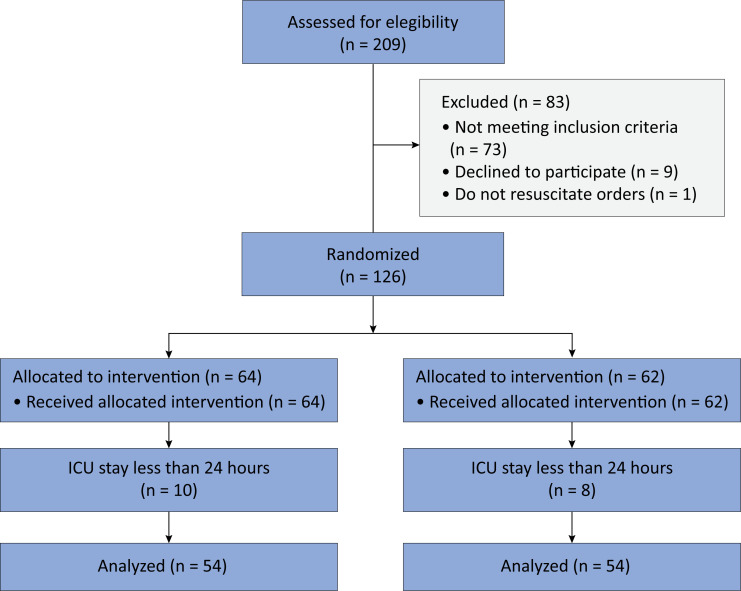
Consolidated Standards of Reporting Trials flowchart of the study.

A total of 209 patients were admitted to the ICU during the study period and were assessed for eligibility. A total of 126 patients were found to be eligible and were randomized into two groups. Ten patients in the Group M and 8 patients in the Group C had an ICU stay of less than 24 hours and were excluded from the analysis. A total of 108 patients (54 in each group) were included in the final analysis (Figure 1). The demographic and baseline data were comparable between the two groups and are described in table 1. The details of the admission diagnoses are provided in table 2.

ICU - intensive care unit.

**Table 1:** Baseline demographic data of the 2 groups

		Group M (n = 54)	Group C (n = 54)
Age (years)		34.7 ± 14.5	35.1 ± 14.2
Gender (M/F)		18/36	15/39
Specialty			
	Medical	29 (53.7)	41 (75.9)
	Surgical	25 (46.3)	13 (24.1)
Admitted from			
	Ward	20 (37)	22 (40.7)
	Emergency	19 (35.2)	23 (42.6)
	Operating room	15 (27.8)	9 (16.7)
APACHE II score		16 (12.75 - 21.25)	16.5 (13 - 20)
*Delirium* at admission to ICU		29 (53.7)	39 (72.2)

M - male; F - female; APACHE - Acute Physiology and Chronic Health Evaluation; ICU - intensive care unit. Results expressed as the mean ± standard deviation, n (%) or median and interquartile range

**Table 2:** Details of admission diagnoses

Admission diagnoses		Group M (n = 54)	Group C (n = 54)
Respiratory			
	Pneumonia	4	5
	Acute exacerbation of asthma	0	2
Obstetrics			
	PPH	6	2
	Preclampsia	5	5
	Pyometra with sepsis	1	2
	Ruptured ectopic/peripartum cardiomyopathy/severe heart disease	0/2/1	2/0/0
Tropical fever			
	Dengue shock syndrome	2	3
	Scrub typhus	1	5
Neurologic/neuromuscular			
	LGBS/neuroparalytic snake bite	3/5	4/0
	Cerebral vascular accident	0	2
	Meningoecephalitis/Myesthenic crisis	0/1	2/0
Partial hanging		1	4
Poisoning			
	Organophosphate poisoning	1	2
	Others	2	2
Gastroenterologic			
	Acute pancreatitis	0	1
	ALF/DCLD	3/1	1/0
	Acute gastroenteritis	0	1
Septic shock		4	3
SLE flare-up		3	0
Diabetic		1	1
Miscellaneous		7	5

PPH - postpartum hemorrhage; LGBS - Landry-Guillain-Barré syndrome; ALF/DCLD - acute liver failure/decompensated chronic liver disease; SLE - systemic lupus erythematosus. Results express as the n.

There was no statistically significant difference in the incidence of *delirium* either in the first 24 hours (primary outcome) or on the third or seventh day after ICU admission (Table 3). Among the subset of patients who were not delirious upon arrival, the incidence of new-onset *delirium* at Days 1, 3, and 7 or at discharge was significantly similar between the two groups (Table 4). Among those who were delirious upon arrival, the proportions of patients who continued to be delirious on Day 1 (51.7% in Group M *versus* 28.2% in Group C), 3 (24.7% *versus* 28.2%) or throughout the 7-day study period/discharge (20.6% *versus* 12.8%) were also significantly different between the two groups.

**Table 3:** Comparison of the incidence of *delirium*, mortality, length of intensive care unit stay, length of mechanical ventilation and Glasgow coma scale score between the 2 groups

	Group M (n = 54)	Group C (n = 54)	Risk ratio (CI95%)	p value
*Delirium* - Day 1	16 (29.6)	25 (46.2)	0.6 (0.38 - 1.05)	0.11
*Delirium* - Day 3	10 (18.5)	14 (25.9)	0.7 (0.34 - 1.46)	0.48
*Delirium* - Day 7/at discharge	9 (16.6)	7 (12.9)	1.3 (0.51 - 3.20)	0.78
Mortality	11 (20.4)	5 (9.3)	2.2 (0.8 - 5.9)	0.10
Length of ICU stay in days	5 (4 - 9)	7.5 (5 - 11.75)	-	0.1
Duration of mechanical ventilation in days	3 (2 - 7)	4.5 (2 - 7.5)	-	0.5
Glasgow coma scale score	5 (4 - 5)	5 (4 - 5)	-	0.5

CI95% - confidence interval 95%; ICU - intensive care unit. Results expressed as the n (%) or median and interquartile range.

**Table 4:** Incidence of new-onset *delirium* in the intensive care unit at various time points

	Group M (n = 25)	Group C (n = 15)	Risk ratio (CI95%)	p value
New onset *delirium* on Day 1	1	2	0.30 (0.02 - 3.03)	0.62
New onset *delirium* on Day 3	2	0	2.4 (0.11 - 49.77)	0.99
New onset *delirium* on Day 7	2	2	0.6 (0.09 - 3.82)	0.96
New onset *delirium* at any time in ICU	3	4	0.45 (0.11 - 1.74)	0.44

CI95% - confidence interval 95%; ICU - intensive care unit.

The daily SOFA score; sodium, potassium, calcium and magnesium levels; and NRS for pain were similar between the groups. The mortality, length of ICU stay, duration of MV and Glasgow outcome scale score were also similar between the 2 groups (Table 3). Ten out of the 108 patients required the use of rescue/additional haloperidol—3 (5.5%) in Group M and 7 (12.9%) in Group C [RR 0.42 (CI95% 0.12 - 1.57); p = 0.31].

## DISCUSSION

In this study, we demonstrated that 3mg of enterally administered melatonin did not decrease the incidence of *delirium* on Days 1, 3 and 7 in the ICU compared to the control. Furthermore, the SOFA score on Day 1 was found to be an independent predictor of *delirium* in patients who were not initially delirious at the time of ICU admission.

Intensive care unit-acquired *delirium* is a common problem encountered by intensivists and is associated with poorer outcomes in terms of mortality, duration of MV, length of ICU stay and hospital costs.^(11–13)^ A variety of factors may lead to the development of *delirium*, including substance abuse, advanced age, pain, electrolyte disturbances, and disturbance of the sleep-wake cycle. A disturbance in the circadian rhythm is an important and independent risk factor for ICU-acquired *delirium*.^(14)^ Sleep deprivation may result from environmental disturbances, loss of diurnal routine, the critical illness itself or the treatment being administered. Routine pharmacological prophylaxis is not recommended for the prevention of *delirium*. Enteral melatonin supplementation decreases circadian disruption and may in turn decrease the incidence of *delirium*. It is inexpensive and has no known serious adverse effects. However, its efficacy in preventing *delirium* has not yet been well established. In their SRMA of 12 trials involving the use of melatonin/ramelteon, Yan et al.^(7)^ reported a significant decrease in the incidence of *delirium* in the experimental group.^(15)^ However, a sensitivity analysis and a trial sequential analysis revealed that this conclusion may be due to a false-positive error. These findings echo those of the more recently published SRMA by Aiello et al.,^(8)^ which included 9 RCTs (6 reported incidences of *delirium*) and did not report any decrease in *delirium* in the experimental group. However, the high level of heterogeneity in both of these SRMA limited the quality of the evidence. Furthermore, many of the trials included in these meta-analyses were conducted on cardiac surgical patients, which is quite unlike the patient population included in this study.^(16–18)^

In our study, the addition of 3mg of enteral melatonin did not have any effect on the incidence of *delirium* throughout the ICU stay. This finding is similar to the results of Abbasi et al.^(19)^ Their incidence of *delirium* was much lower than ours, at 4.5% and 1.4% in the melatonin and placebo groups, respectively. Naderi-Behdani et al., in their RCT investigating the effect of melatonin on blood sugar fluctuations and insulin resistance, did not demonstrate any decrease in *delirium* (one of the secondary outcomes) with melatonin administration.^(20)^ A recently published multicenter RCT by Wibrow et al. also failed to demonstrate any decrease in *delirium*-free days with the administration of 4mg of enteral melatonin compared to placebo in critically ill adults.^(21)^ On the other hand, Baumgartner et al. reported a significantly lower occurrence of *delirium* with melatonin than with placebo.^(22)^ However, this was a retrospective observational study inherently limited by recall bias and possibly inadequate data collection. Vijayakumar et al. also reported a decrease in *delirium* with melatonin in patients with organophosphate poisoning.^(23)^ Notably, organophosphate poisoning and its treatment with atropine result in a high incidence of *delirium*. Additionally, their primary outcome was the duration of *delirium*, and their sample size was calculated based upon an unpublished pilot study; thus, their study may be underpowered for determining the prevalence of *delirium*. These findings have not been reproduced in subsequent studies by Bellapart et al. and Gandolfi et al., which also did not include the prevalence of *delirium* as a primary outcome.^(24,25)^

Dianatkhah et al. reported a decreased length of ICU stay, length of MV and mortality in 40 intubated hemorrhagic stroke patients treated with high-dose melatonin (30mg).^(26)^ However, these large doses have not been studied extensively and are currently not deemed safe. These results were not reproduced in further larger RCTs, such as Abbasi et al., Gandolfi et al. or our study.^(19,25)^

In our study, there was strict adherence to the study protocol; all patients randomized to the Group M actually received the drug. Moreover, there was no loss to follow-up, and all patients completed the 7-day trial period. Second, our sample size calculation was robust because it was based upon already published literature regarding the incidence of *delirium* in our ICU. Finally, our study involved a mixed medical-surgical ICU with a good mix of patients, which lends adequate generalizability of the results.

Our study was not without limitations. First, ours was not a placebo-controlled trial, which could have introduced caregiver bias. The outcome assessor was blinded to the treatment allocation to reduce observer bias. Second, despite hypothesizing a decrease in the disruption of the circadian rhythm and improvement in sleep quality as the putative mechanism of a reduction in the incidence of *delirium*, we did not assess the sleep quality of the patients. However, we provided all patients with adequate conditions to promote night-time sleep and kept patients oriented during the day hours to maintain the circadian rhythm. Furthermore, *delirium* is a multifactorial disorder with complex underlying mechanisms, and addressing only one such mechanism (disruption of circadian rhythm) may not be enough to determine the effect size we initially aimed for in this study.

## CONCLUSION

Our study showed that enterally administered melatonin at a dose of 3mg once a day for one week did not reduce the incidence of *delirium* in critically ill adults admitted to the intensive care unit. There was no reduction in intensive care unit mortality, length of intensive care unit stay, duration of mechanical ventilation or Glasgow coma scale score at discharge in the Melatonin Group compared to the Control Group. More work needs to be done with different melatonin doses, in different intensive care unit settings and on other population subgroups to further our research findings.
