# Supplementary material for: Efficacy of melatonin in decreasing the incidence of delirium in critically ill adults: a randomized controlled trial
Source: Crit Care Sci. 2024 Apr 9;36:e20240144en. doi: 10.62675/2965-2774.20240144-en (PMC11098074; doi:10.62675/2965-2774.20240144-en)
Supplement: Translation

# Translation: Eficácia da melatonina na redução da incidência de *delirium* em pacientes adultos graves: um ensaio clínico randomizado

**DOI:** 10.62675/2965-2774.20240144-pt

**Authors:** Anjishnujit Bandyopadhyay, Lakshmi Narayana Yaddanapudi, Vikas Saini, Neeru Sahni, Sandeep Grover, Sunaakshi Puri, Vighnesh Ashok

**Affiliations:** 1 Nova Delhi Índia Department of Anaesthesiology, Pain Medicine and Critical Care, Jai Prakash Narayan Apex Trauma Center, All India Institute of Medical Sciences - Nova Delhi, Índia.; 2 Chandigar Índia Department of Anaesthesia and Intensive Care, Level 4, Nehru Hospital, Postgraduate Institute of Medical Education and Research, Sector 12 - Chandigar, Índia.; 3 Chandigar Índia Department of Psychiatry, Nehru Hospital, Postgraduate Institute of Medical Education and Research, Sector 12 - Chandigar, Índia.

**Keywords:** Ritmo circadiano, Estado terminal, Delirium, Melatonina, Transtornos do sono-vigília, Unidades de terapia intensiva

## Abstract

**Objetivo:**

Determinar se a melatonina enteral diminui a incidência de *delirium* em adultos em estado grave.

**Métodos:**

Neste estudo controlado e randomizado, os adultos foram admitidos à unidade de terapia intensiva e/ou receberam apenas o padrão de cuidado habitual (Grupo Controle) ou o tratamento combinado com 3mg de melatonina enteral uma vez ao dia às 21h (Grupo Melatonina). A ocultação da alocação foi feita por meio de envelopes selados opacos e numerados sequencialmente. O intensivista que avaliou o delirium e o pesquisador que realizou a análise dos dados foram cegados quanto à alocação do grupo. O desfecho primário foi a incidência de *delirium* dentro de 24 horas de internação na unidade de terapia intensiva. Os desfechos secundários foram a incidência de *delirium* nos dias 3 e 7, a mortalidade na unidade de terapia intensiva, a duração da internação na unidade de terapia intensiva, a duração da ventilação mecânica e o escore da escala de desfecho de Glasgow (na alta).

**Resultados:**

Foram incluídos 108 pacientes na análise final, com 54 sujeitos em cada grupo. Em 24 horas de internação na unidade de terapia intensiva, a incidência de *delirium* não foi diferente entre os Grupos Melatonina e Controle (29,6% *versus* 46,2%; RR = 0,6; IC95% 0,38 - 1,05; p = 0,11). Nenhum desfecho secundário apresentou diferenças estatisticamente significativas.

**Conclusão:**

Em adultos em estado grave, 3mg de melatonina enteral não foi mais eficaz que os cuidados padrão na redução da incidência de *delirium*.

## INTRODUÇÃO

O *delirium* é um estado confusional agudo caracterizado por distorções flutuantes na cognição e na atenção. Sabe-se que está associado à desregulação da neurotransmissão dopaminérgica, colinérgica, gama-aminobutírica (GABAérgica), serotoninérgica e catecolíngica.^(1)^ A prevalência de *delirium* varia entre 11 e 83% em pacientes internados na unidade de terapia intensiva (UTI).^(2,3)^ O *delirium* aumenta o risco de mortalidade, o tempo de internação na UTI e os custos hospitalares e, desse modo, merece tanta atenção quanto outras falências orgânicas que acontecem nessa unidade.^(4)^

A etiologia do *delirium* é multifatorial e, em um ambiente de UTI, a interrupção do ritmo circadiano e do ciclo do sono pode ser um importante fator relacionado. A privação do sono na UTI ocorre devido a interferências ambientais, alteração da rotina diurna, pela própria gravidade da doença ou pelo tratamento administrado aos pacientes.^(5)^

A justificativa para o uso da melatonina na redução dos distúrbios do sono e, possivelmente, do *delirium* na UTI deriva da constatação de baixas concentrações plasmáticas de melatonina e de padrões anormais de secreção de melatonina em pacientes graves.^(6)^ Contudo, ainda não foi estabelecido um efeito profilático e nem terapêutico definitivo da melatonina, como fica evidente pelos achados divergentes relatados em revisões sistemáticas e metanálises publicadas por Yan et al. e Aiello et al.^(7,8)^

Neste estudo, avaliamos se a melatonina profilática ou terapêutica diminui a incidência de *delirium* em comparação com o padrão de cuidado em adultos em estado grave internados na UTI. A duração da ventilação mecânica (VM), o tempo de internação na UTI, a mortalidade e o escore da escala de desfecho de Glasgow na alta foram estudados como desfechos secundários. Nosso objetivo foi, pois, determinar se a melatonina enteral diminui a incidência de *delirium* em adultos em estado grave.

## MÉTODOS

Trata-se de um estudo controlado randomizado aberto com grupos paralelos realizado em uma UTI mista médico-cirúrgica de um hospital universitário de Atenção Terciária. O estudo foi realizado entre janeiro de 2020 e outubro de 2020, após o registro no *Clinical Trials Registry* (CTRI/2020/01/023038) da Índia e aprovação do Comitê de Ética institucional. Foram triados para inclusão todos os pacientes entre 18 e 65 anos de idade com previsão de internação na UTI por mais de 24 horas. O Termo de Consentimento Livre e Esclarecido foi obtido dos pacientes ou responsáveis antes do recrutamento para o estudo. Foram excluídos do estudo pacientes gestantes, já em tratamento com melatonina, comatosos que não puderam ser avaliados quanto ao *delirium*, com a doença pelo coronavírus 2019 (COVID-19), já em tratamento com medicamentos antipsicóticos, com doença de Alzheimer ou doença de Parkinson e aqueles para os quais era contraindicada a administração de medicamentos enterais.

Os pacientes foram alocados aleatoriamente em um dos dois grupos usando a randomização permutada em blocos gerada por computador. A ocultação da alocação foi feita por meio de envelopes selados opacos e numerados sequencialmente. O tratamento foi revelado somente quando o paciente foi recrutado para o estudo e antes da primeira administração do medicamento. O intensivista que avaliou o *delirium* e o pesquisador que realizou a análise dos dados foram cegados quanto à alocação do grupo.

Os pacientes do Grupo Melatonina (Grupo M) receberam 3mg de melatonina por via enteral às 21h por 7 dias consecutivos, além do padrão de cuidado ou até a alta da UTI, o que ocorresse primeiro. Os pacientes do Grupo Controle (Grupo C) receberam apenas o padrão de cuidado. O padrão de cuidado habitual à prevenção do *delirium* inclui evitar sedação e restrições desnecessárias, suspensão da sedação, maior interação com a família, mobilidade e exercícios precoces, manejo adequado da dor e prevenção da hipóxia e da diseletrolitemia. O uso de sedoanalgesia foi deixado a critério de um intensivista de plantão, mas evitaram-se benzodiazepínicos intravenosos, conforme protocolo da UTI. Os medicamentos comumente usados para a sedoanalgesia em nossa UTI são a dexmedetomidina e o fentanil. A infusão de sedação foi direcionada com base em um escore da escala de agitação e sedação de Richmond (RASS) de zero a um.

Imediatamente após a admissão e o sucesso no recrutamento do estudo, foram coletados dados basais, que incluíam idade, sexo, diagnóstico no momento da admissão, escore da escala de desfecho de Glasgow, escore da *Acute Physiology and Chronic Health Evaluation* (APACHE II) e eletrólitos séricos (sódio, potássio, cálcio e magnésio). Os escores *Sequential Organ Failure Assessment* (SOFA), eletrólitos séricos e escores de classificação numérica (NRS - *numerical rating scores*) de dor foram medidos diariamente.

Realizou-se uma avaliação de *delirium* por meio do método de avaliação de confusão (CAM) na UTI por um intensivista que não participou do estudo. O CAM na UTI avalia os pacientes em relação a quatro aspecto: flutuações do estado mental, desatenção, alteração do nível de consciência e desorganização do pensamento.^(9)^ No caso de agitação aguda, quando havia risco de extubação ou falha no desmame, utilizou-se uma dose de 0,5mg de haloperidol intravenoso (IV) a cada 30 minutos como agente de resgate até que a agitação diminuísse ou até que se atingisse uma dose de 2,5mg, ocasião em que se administravam 2mg de lorazepam IV. Se o paciente continuasse agitado, solicitava-se uma consulta psiquiátrica de emergência. Os pacientes que desenvolveram *delirium* após o período de 7 dias foram tratados com 0,25mg de haloperidol IV duas vezes ao dia, seguindo o protocolo da UTI. Os pacientes foram seguidos diariamente, e foram registrados os seguintes dados: duração da VM, tempo de internação na UTI, necessidade e dose de agentes sedativos ou antipsicóticos, efeitos adversos, caso houvesse, devido à melatonina, mortalidade e escala de desfecho de Glasgow.

O desfecho primário foi a incidência de *delirium* (proporção de pacientes que estavam delirantes na avaliação) dentro de 24 horas de internação na UTI em ambos os grupos. Os desfechos secundários foram a incidência de *delirium* nos dias 3 e 7 da internação na UTI; incidência de *delirium* (*delirium* de início recente) nas 24 horas nos dias 3 e 7; mortalidade; tempo de internação na UTI; tempo de VM; e escore da escala de desfecho de Glasgow na alta.

Um estudo anterior realizado em nossa UTI mostrou 68% de prevalência e 60% de incidência de *delirium*.^(10)^ O cálculo do tamanho da amostra foi realizado no site www.openepi.com. Para obter redução de 50% na prevalência de *delirium* no grupo de tratamento, foram recrutados 49 pacientes em cada grupo, com poder de 90% e erro alfa de 5%. Pretendíamos recrutar 126 pacientes em cada grupo, contabilizando 25% de desistências durante o período do estudo. Os dados categóricos, como prevalência e mortalidade, foram analisados usando o teste do qui-quadrado ou o teste exato de Fisher e são apresentados como índices de risco com intervalos de confiança de 95% (IC95%). A normalidade dos dados foi analisada com o teste de Shapiro-Wilk. Os dados que seguem uma distribuição gaussiana, como a idade, são apresentados como média e desvio-padrão e foram analisados com o teste *t* de Student. Os dados não gaussianos, como tempo de internação na UTI, VM e APACHE II, são apresentados como mediana e intervalo interquartil e foram analisados com o teste U de Mann-Whitney. Todos os testes foram bicaudais, com IC95% e nível de significância de 5% (p < 0,05). Essa análise foi realizada com o Jamovi versão 1.6 baseado na versão 4.0 do R Core.

## RESULTADOS

Foram admitidos 209 pacientes na UTI durante o período do estudo, os quais foram avaliados para inclusão no estudo. Foram considerados aptos 126 pacientes, os quais foram distribuídos aleatoriamente em dois grupos. Dez pacientes no Grupo M e oito pacientes no Grupo C permaneceram na UTI por menos de 24 horas e foram excluídos da análise. A análise final incluiu 108 pacientes (54 em cada grupo) (Figura 1). Os dados demográficos e basais foram comparáveis entre os dois grupos e estão descritos na tabela 1. Os detalhes dos diagnósticos de admissão são fornecidos na tabela 2.

**Figura 1:** Fluxograma do estudo do *Consolidated Standards of Reporting Trials*.

UTI - unidade de terapia intensiva.

**Tabela 1:** Dados demográficos basais dos dois grupos

		Grupo M (n = 54)	Grupo C (n = 54)
Idade (anos)		34,7 ± 14,5	35,1 ± 14,2
Sexo (M/F)		18/36	15/39
Especialidade			
	Médica	29 (53,7)	41 (75,9)
	Cirúrgica	25 (46,3)	13 (24,1)
Admitido da			
	Enfermaria	20 (37)	22 (40,7)
	Emergência	19 (35,2)	23 (42,6)
	Sala de cirurgia	15 (27,8)	9 (16,7)
APACHE II		16 (12,75 - 21,25)	16,5 (13 - 20)
*Delirium* na admissão à UTI		29 (53,7)	39 (72,2)

M - masculino; F - feminino; APACHE - *Acute Physiology and Chronic Health Evaluation*; UTI - unidade de terapia intensiva. Resultados expressos como média ± desvio-padrão, n (%) ou mediana e intervalo interquartil.

**Tabela 2:** Detalhes dos diagnósticos de admissão

Diagnósticos de admissão		Grupo M (n = 54)	Grupo C (n = 54)
Respiratório			
	Pneumonia	4	5
	Exacerbação aguda da asma	0	2
Obstetrícia			
	HPP	6	2
	Pré-eclâmpsia	5	5
	Piometra com sepse	1	2
	Ruptura de cardiomiopatia/doença cardíaca grave ectópica/periparto	0/2/1	2/0/0
Febre tropical			
	Síndrome do choque da dengue	2	3
	Tifo esfoliante	1	5
Doenças neurológicas/neuromusculares			
	SLGB/neuroparalisia por picada de cobra	3/5	4/0
	Acidente vascular cerebral	0	2
	Meningoecefalite/crise miestênica	0/1	2/0
Enforcamento parcial		1	4
Intoxicação			
	Intoxicação por organofosfato	1	2
	Outros	2	2
Doenças gastroenterológicas			
	Pancreatite aguda	0	1
	IHA/DCLD	3/1	1/0
	Gastroenterite aguda	0	1
Choque séptico		4	3
Surto de LES		3	0
Diabético		1	1
Diversos		7	5

HPP - hemorragia pós-parto; SLGB - síndrome de Landry-Guillain-Barré; IHA/DCLD - insuficiência hepática aguda/doença hepática crônica descompensada; LES - lúpus eritematoso sistêmico. Resultados expressos como n.

Não houve diferença estatisticamente significativa na incidência de *delirium* nas primeiras 24 horas (desfecho primário) ou nos dias 3 e 7 após a admissão na UTI (Tabela 3). No subgrupo de pacientes que não apresentaram *delirium* na admissão, a incidência de *delirium* de início recente foi significativamente semelhante entre os dois grupos nos dias 1, 3 e 7 ou na alta (Tabela 4). Entre aqueles que estavam delirantes na admissão, as proporções de pacientes que continuaram delirantes no dia 1 (51,7% no Grupo M *versus* 28,2% no Grupo C), no dia 3 (24,7% *versus* 28,2%) ou durante todo o período de estudo de 7 dias/alta (20,6% *versus* 12,8%) também foram significativamente diferentes entre os dois grupos.

**Tabela 3:** Comparação da incidência de *delirium*, mortalidade, tempo de internação na unidade de terapia intensiva, duração da ventilação mecânica e escore da escala de coma de Glasgow entre os dois grupos

	Grupo M (n = 54)	Grupo C (n = 54)	Razão de risco (IC95%)	Valor de p
*Delirium*- dia 1	16 (29,6)	25 (46,2)	0,6 (0,38 - 1,05)	0,11
*Delirium*- dia 3	10 (18,5)	14 (25,9)	0,7 (0,34 - 1,46)	0,48
*Delirium*- dia 7/na alta	9 (16,6)	7 (12,9)	1,3 (0,51 - 3,20)	0,78
Mortalidade	11 (20,4)	5 (9,3)	2,2 (0,8 - 5,9)	0,10
Tempo de internação na UTI em dias	5 (4 - 9)	7,5 (5 - 11,75)	-	0,1
Duração da ventilação mecânica em dias	3 (2 - 7)	4,5 (2 - 7,5)	-	0,5
Escore de escala de coma de Glasgow	5 (4 - 5)	5 (4 - 5)	-	0,5

IC95% - intervalo de confiança de 95%; UTI - unidade de terapia intensiva. Resultados expressos como n (%) ou mediana e intervalo interquartil.

**Tabela 4:** Incidência de *delirium* de início recente na unidade de terapia intensiva em vários momentos

	Grupo M (n = 25)	Grupo C (n = 15)	Razão de risco (IC95%)	Valor de p
*Delirium* de início recente no dia 1	1	2	0,30 (0,02 - 3,03)	0,62
*Delirium* de início recente no dia 3	2	0	2,4 (0,11 - 49,77)	0,99
*Delirium* de início recente no dia 7	2	2	0,6 (0,09 - 3,82)	0,96
*Delirium* de início recente a qualquer momento na UTI	3	4	0,45 (0,11 - 1,74)	0,44

IC95% - intervalo de confiança de 95%; UTI - unidade de terapia intensiva.

O SOFA diário, os níveis de sódio, potássio, cálcio e magnésio e o NRS de dor foram semelhantes entre os grupos. A mortalidade, o tempo de internação na UTI, a duração da VM e o escore da escala de desfecho de Glasgow também foram semelhantes entre os dois grupos (Tabela 3). Dos 108 pacientes, dez necessitaram usar haloperidol de resgate/adicional, sendo três (5,5%) no Grupo M e sete (12,9%) no Grupo C (razão de risco de 0,42 (IC95% 0,12 - 1,57); p = 0,31).

## DISCUSSÃO

Neste estudo, demonstramos que 3mg de melatonina administrada por via enteral não diminuíram a incidência de *delirium* nos dias 1, 3 e 7 na UTI em comparação com o controle. Além disso, o SOFA no dia 1 foi considerado preditor independente de *delirium* em pacientes que não estavam inicialmente com *delirium* no momento da admissão na UTI.

O *delirium* adquirido na unidade de terapia intensiva é um problema comum encontrado pelos intensivistas e está associado a desfechos mais desfavoráveis em termos de mortalidade, duração da VM, tempo de internação na UTI e custos hospitalares.^(11–13)^ Vários fatores podem levar a um quadro de *delirium*, incluindo abuso de substâncias, idade avançada, dor, distúrbios eletrolíticos e distúrbios do ciclo sono-vigília. Uma interrupção no ritmo circadiano é um fator de risco importante e independente no *delirium* adquirido na UTI.^(14)^ A privação do sono pode resultar de interferências ambientais, alteração da rotina diurna, da própria gravidade da doença ou do tratamento administrado. Não se recomenda a profilaxia farmacológica de rotina para a prevenção do *delirium*. A suplementação de melatonina por via enteral diminui a interrupção do ritmo circadiano e pode, por sua vez, diminuir a incidência de *delirium*. Ela é acessível e não apresenta efeitos adversos graves conhecidos. Entretanto, sua eficácia na prevenção do *delirium* ainda não foi bem estabelecida. Em sua revisão sistemática e metanálise com 12 estudos sobre o uso de melatonina ou ramelteona, Yan et al.^(7)^ relataram diminuição significativa na incidência de *delirium* no grupo experimental.^(15)^ No entanto, uma análise de sensibilidade e uma análise sequencial de estudos revelaram que essa conclusão pode ser devida a um erro falso-positivo. Esses achados coincidem com os da revisão sistemática e metanálise publicada recentemente por Aiello et al.,^(8)^ a qual incluiu nove ensaios clínicos randomizados (seis relataram incidências de *delirium*) e não relatou diminuição de *delirium* no grupo experimental. Entretanto, o alto nível de heterogeneidade em ambas as metanálises limitou a qualidade das evidências. Além disso, muitos dos estudos incluídos nessas metanálises foram realizados em pacientes de cirurgia cardíaca, o que difere completamente da população de pacientes incluída neste estudo.^(16–18)^

Em nosso estudo, a administração de 3mg de melatonina enteral não teve efeito sobre a incidência de *delirium* durante a internação na UTI. Esse achado é semelhante aos resultados de Abbasi et al.^(19)^ Sua incidência de *delirium* foi muito menor do que a nossa, com 4,5% e 1,4% nos grupos melatonina e placebo, respectivamente. Naderi-Behdani et al., em seu ensaio clínico randomizado que investigou o efeito da melatonina nas variações de glicemia e na resistência à insulina, não demonstraram diminuição do *delirium* (um dos desfechos secundários) com a administração de melatonina.^(20)^ Um ensaio clínico randomizado multicêntrico publicado recentemente por Wibrow et al. também não conseguiu demonstrar diminuição nos dias sem *delirium* com a administração de 4mg de melatonina enteral em comparação com placebo em adultos graves.^(21)^ Por outro lado, Baumgartner et al. relataram uma ocorrência significativamente menor de *delirium* com melatonina do que com placebo.^(22)^ No entanto, esse foi um estudo observacional retrospectivo inerentemente limitado pelo viés de recordação e possivelmente pela coleta inadequada de dados. Vijayakumar et al. também relataram diminuição do *delirium* com melatonina em pacientes com intoxicação por organofosfato.^(23)^ Em particular, a intoxicação por organofosfato e seu tratamento com atropina resultam em grande incidência de *delirium*. Além disso, seu desfecho primário foi a duração do *delirium*, e o tamanho da amostra foi calculado como base em um estudo-piloto não publicado; portanto, seu estudo pode não ter poder suficiente para determinar a prevalência de *delirium*. Esses achados não foram reproduzidos em estudos subsequentes realizados por Bellapart et al. e Gandolfi et al., que também não incluíram a prevalência de *delirium* como desfecho primário.^(24,25)^

Dianatkhah et al. relataram menor tempo de internação na UTI, duração da VM e mortalidade em 40 pacientes com acidente vascular cerebral hemorrágico intubados e tratados com altas doses (30mg) de melatonina.^(26)^ No entanto, essas doses elevadas não foram estudadas extensivamente e atualmente não são consideradas seguras. Esses resultados não foram reproduzidos em outros ensaios clínicos randomizados mais amplos, como os de Abbasi et al., Gandolfi et al. ou no nosso estudo.^(19,25)^

Em nosso estudo, observou-se uma adesão rigorosa ao protocolo do estudo. Todos os pacientes randomizados ao Grupo M realmente receberam o medicamento. Além disso, não houve perda de seguimento e todos os pacientes concluíram o período experimental de 7 dias. Em segundo lugar, nosso cálculo do tamanho da amostra foi robusto porque se baseou na literatura já publicada sobre a incidência de *delirium* em nossa UTI. Por fim, nosso estudo envolveu uma UTI mista médico-cirúrgica com uma boa combinação de pacientes, o que permite uma generalização adequada dos resultados.

Nosso estudo não foi isento de limitações. Em primeiro lugar, não foi um estudo controlado por placebo, o que poderia ter introduzido o viés do cuidador. O avaliador do desfecho não tinha conhecimento da alocação do tratamento para reduzir o viés do observador. Em segundo lugar, apesar da hipótese de uma diminuição na interrupção do ritmo circadiano e melhora na qualidade do sono como o mecanismo putativo de uma diminuição na incidência de *delirium*, não avaliamos a qualidade do sono dos pacientes. Entretanto, fornecemos a todos os pacientes condições adequadas para promover o sono noturno e mantivemos os pacientes orientados durante o dia para manter o ritmo circadiano. Além disso, o *delirium* é um distúrbio multifatorial com mecanismos subjacentes complexos, e a abordagem de apenas um desses mecanismos (interrupção do ritmo circadiano) pode não ser suficiente para determinar o tamanho do efeito que inicialmente almejávamos neste estudo.

## CONCLUSÃO

Nosso estudo revelou que a melatonina administrada por via enteral na dose de 3mg uma vez ao dia durante uma semana não reduziu a incidência de *delirium* em adultos graves internados na unidade de terapia intensiva. Não houve diminuição da mortalidade na unidade de terapia intensiva, do tempo de internação na unidade de terapia intensiva, da duração da ventilação mecânica ou do escore da escala de desfecho de Glasgow na alta no Grupo Melatonina em comparação com o Grupo Controle. É necessário realizar mais estudos com diferentes doses de melatonina, em diferentes ambientes de unidades de terapia intensiva e em outros subgrupos populacionais para aprofundar os achados de nosso estudo.
